# Trends and socioeconomic inequalities in cancer survival in England and Wales up to 2001

**DOI:** 10.1038/sj.bjc.6601696

**Published:** 2004-03-09

**Authors:** M P Coleman, B Rachet, L M Woods, E Mitry, M Riga, N Cooper, M J Quinn, H Brenner, J Estève

**Affiliations:** 1 Cancer and Public Health Unit, London School of Hygiene and Tropical Medicine, Keppel Street, London WC1E 7HT, UK; 2 Health and Care Division, Office for National Statistics, 1 Drummond Gate, London SW1V 2QQ, UK; 3 Abt Epidemologie, Deutsches Zentrum für Alternsforschung (DZFA), Bergheimer Str. 20, D-Heidelberg 69115, Germany; 4 Biostatistics Division, Hospices Civils de Lyon, Université Claude Bernard, 162 avenue Lacassagne, F-69424 Lyon Cédex 3, France

**Keywords:** relative survival, deprivation, socioeconomic inequalities, England and Wales

## Abstract

We examined national trends and socioeconomic inequalities in cancer survival in England and Wales during the 1990s, using population-based data on 2.2 million patients who were diagnosed with one of the 20 most common cancers between 1986 and 1999 and followed up to 2001. Patients were assigned to one of five deprivation categories (from ‘affluent’ to ‘deprived’) using characteristics of their electoral ward of residence at diagnosis. We estimated relative survival up to 5 years after diagnosis, adjusting separately in each deprivation category for background mortality by age, sex and calendar period. We estimated trends in survival and in the difference in survival between deprivation categories (‘deprivation gap’) over the periods 1986–90, 1991–95 and 1996–99. We used period analysis to examine likely survival rates in the near future. Survival improved for most cancers in both sexes during the 1990s, and appears likely to continue improving for most cancers in the near future. The deprivation gap in survival between rich and poor was wider for patients diagnosed in the late 1990s than in the late 1980s. Increases in cancer survival in England and Wales during the 1990s are shown to be significantly associated with a widening deprivation gap in survival.

Cancer survival in England and Wales depends on socioeconomic status. Among adults living in the most deprived areas who were diagnosed during 1981–90, 5-year survival was significantly lower than for those in the most affluent areas for 44 of 47 different cancers (Coleman *et al*, 1999).

Strategic changes in cancer management from 1995 were designed to improve cancer outcomes and the equality of access to optimal cancer care (Expert Advisory Group on Cancer, 1995). Government policy to reduce delays in diagnosis and treatment (NHS Executive, 1997) would also be expected to lead to higher survival rates in due course (Stockton *et al*, 1997; Richards *et al*, 1999).

We have examined national trends and socioeconomic inequalities in cancer survival in England and Wales for patients diagnosed up to 1999 and followed up to 31 December 2001.

## MATERIALS AND METHODS

We examined the data for some 2.2 million patients diagnosed aged 15–99 years with one of the 20 most common cancers between 1 January 1986 and 31 December 1999 in England and Wales (Table 1

**Table 1:** Cancer patients diagnosed in England and Wales, 1986–99: exclusions (% of those eligible) and number (%) of eligible cases included in survival analysis

**Malignancy**	**Zero survival^a^**	**Multiple primary^b^**	**Other^c^**	**Analysed no.**	**%**
Oesophagus	10.8	3.6	1.7	65 591	83.9
Stomach	14.2	3.0	2.0	112 367	80.8
Colon	11.5	3.5	2.4	206 879	82.6
Rectum	6.6	3.4	2.2	132 602	87.8
Pancreas	22.0	2.8	1.7	62 815	73.6
Larynx	4.3	3.8	2.8	20 112	89.2
Lung	16.0	3.2	2.0	391 678	78.8
Melanoma	2.8	3.0	2.7	55 394	91.5
Breast (F)	6.1	2.4	3.1	382 277	88.5
Cervix	3.9	3.1	3.1	44 090	89.9
Uterus	5.7	5.2	2.2	53 092	87.0
Ovary	10.2	3.6	2.0	63 833	84.2
Prostate	8.5	3.3	1.9	201 134	86.3
Testis	0.9	0.9	2.8	18 605	95.4
Bladder	5.2	4.4	2.3	141 531	88.1
Kidney	12.2	4.6	2.0	49 721	81.3
Brain	9.8	1.8	2.5	37 917	85.9
Non-Hodgkin lymphoma	9.0	3.2	2.3	78 894	85.5
Myeloma	13.1	3.1	2.0	32 419	81.9
Leukaemia	15.9	3.5	2.3	56 914	78.3
					
Total	10.4	3.2	2.3	2 207 865	84.1

a Date of diagnosis same as date of death: some patients did die on the day of diagnosis, but most were registered solely from death certificate, with unknown survival time.

b Persons who had a previous primary malignancy.

c Aged 100 years or over at diagnosis, vital status or sex unknown, sex-site error, invalid dates, duplicate registration or synchronous tumour.

). Anonymised data were obtained from the Office for National Statistics, after linkage of incident cases in the National Cancer Registry with information on deaths from the NHS Central Register (Office for National Statistics, 2003a). The data were extracted for analysis on 5 November 2002, when the vital status (alive, emigrated, dead, not traced) at 31 December 2001 was known for 98.4% of patients. (The survival of 2 887 690 adults diagnosed 1971–90 and followed up to 1995 was reported in 1999 (Coleman *et al*, 1999). In late 2002, we sought the last known vital status up to 31 December 2001 for the 448 598 patients not known to have died by 31 December 1995. The National Cancer Registry is continuously updated, and in the 5 years since data were extracted for the previous analyses in July 1997, the regional cancer registries had requested deletion of 4196 records: 2206 cases in which the diagnosis of cancer had been revoked were excluded, but the corrected tumour record was used for 1990 cases that had been resubmitted after correction of errors in the original data, often with a revised date of birth or diagnosis. Consequently, the number of cases included and the survival estimates for patients diagnosed 1986–90 may differ slightly from those published previously.) Ten percent of patients were excluded because their recorded survival time was zero (mainly death certificate only (DCO) cases whose survival time was unknown) (Table 1). Other patients were excluded because it was not their first cancer (3.2%) or for other reasons (2.3%) including unknown vital status.

Each patient was assigned to one of five categories of socioeconomic deprivation based on their electoral ward of residence at diagnosis, the smallest geographic unit for which adequate data were available over the entire period 1986–99. Deprivation categories were labelled from least deprived (affluent, or ‘rich’) to most deprived (‘poor’). For patients diagnosed during 1986–95, the categories were based on quintiles of the national distribution of the ward Carstairs score (Carstairs, 1995) derived from the 1991 Census. For patients diagnosed during 1996–99, deprivation categories were based on quintiles of the ward income domain score, a subcomponent of the indices of multiple deprivation (IMD) 2000 for England (Department of the Environment Transport and the Regions, 2000), combined with the comparable but not precisely equivalent index available for Wales (National Assembly for Wales, 2000).

Complete life tables by single year of age (up to 99 years), sex and deprivation category were derived from the numbers of deaths in each ward in England and Wales for the periods 1990–92 and 1997–99. Population denominators for the 1990–92 life tables were derived from the 1991 census (Office for National Statistics, 1991). For 1997–99, the age–sex structure of each Local Authority in the 2001 Census (Office for National Statistics, 2003b) was applied to the estimated population counts in 1998 (Penhale and Noble, 2000) for each of its constituent wards (Woods *et al*., submitted for publication).

We estimated national relative survival rates for England and Wales for each cancer and each sex, by age, deprivation category and calendar period of diagnosis. Relative survival is the ratio of the observed (absolute) survival of the cancer patients and the survival that would have been expected if the patients had had the same age- and sex-specific mortality in each period (background mortality) as the general population (Berkson and Gage, 1950). The 1990–92 life tables were used to represent background mortality by age, sex and deprivation category during 1986–95, and the 1997–99 life tables for the period 1996–2001. Survival probabilities for most cancers were estimated at monthly intervals for the first 6 months, then 3-monthly up to 1 year and 6-monthly from 1 to 5 years. Survival for patients diagnosed during 1986–90 was used as the baseline for estimating trends in both national survival rates and deprivation gradients in survival. Cumulative relative survival up to 5 years after diagnosis was estimated separately for patients diagnosed in the calendar periods 1986–90, 1991–95 and 1996–99. For the first two periods, data on at least 5 years of follow-up were available for all patients, and classical cohort analysis of survival was used. For the period 1996–99, 5 years' potential follow-up was only available for those diagnosed in 1996, but the most up-to-date estimates for shorter-term survival probabilities were used for patients diagnosed in later years (complete analysis).

We applied the maximum-likelihood approach for individual records (Estève *et al*, 1990) to estimate observed and relative survival, using the algorithm developed for previous analyses (Coleman *et al*, 1999), modified to enable period analysis (Brenner and Gefeller, 1996). Period analysis is a recent methodological development that enables predictions of, say, 5-year survival to be made for patients who were diagnosed less than 5 years ago; this is analogous to the prediction of life expectancy at birth in a given year from the death rates at all ages observed during that year. We used this approach for the period 2000–01. For this purpose, we estimated the conditional probabilities of relative survival up to 5 years after diagnosis from the survival experience during 2000–01 of cancer patients diagnosed during 1996–99.

Survival gradients across the five categories of deprivation were estimated with linear regression, weighted by the variance of the relative survival estimate (Grizzle *et al*, 1969), using STATA software (StataCorp, 1997). The difference between the relative survival rates fitted by the regression model for the most affluent and most deprived categories is described as the ‘deprivation gap’ in survival. The deprivation gap is reported as negative if survival was lower in the poor than the rich. Changes in deprivation gradient between successive calendar periods, adjusted for secular trends in survival, were estimated from the interaction between calendar period and deprivation. We report the average change in the deprivation gap every 5 years, taking account of the shorter final period (4 years). We also report the temporal change in survival every 5 years, adjusted for change in the deprivation gap.

The relationship between trends in 1- and 5-year survival during the 1990s and concurrent trends in the deprivation gap in survival was also examined, using linear regression weighted by the conditional variance of the change in deprivation gap given the change in survival. The regression was carried out separately for men (16 cancers) and women (17 cancers).

## RESULTS

**Figure 1:**
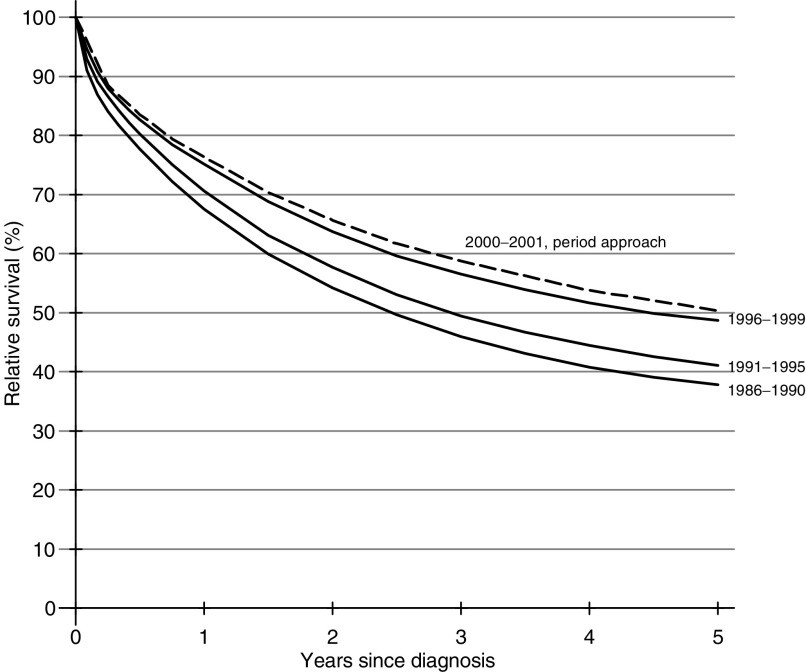
Relative survival up to 5 years, by calendar period of diagnosis, England and Wales, men with cancer of the rectum diagnosed 1986–99 and followed up to 2001. The dashed curve is derived from period analysis using survival experience during 2000–01.

Cancer survival improved steadily for most cancers in both sexes up to 2001. The survival curves for rectal cancer in men diagnosed in successive calendar periods up to 1999 are shown as an example (Figure 1

): the period curve, based on survival experienced during 2000–01, suggests the improvement is still continuing. Five year survival increased for 15 of the 16 cancers examined among men diagnosed in England and Wales over the period 1986–99, and the average increase every 5 years during the 1990s was statistically significant for 11 of these, but not for cancers of the pancreas, lung, testis or bladder (Table 2

**Table 2:** Five-year relative survival (%) and deprivation gap in survival, cancer patients diagnosed in England and Wales 1996–99, with average change every 5 years during 1986–99, and period estimate of 5-year survival (%), using events during 2000–01

	**5-year relative survival (%)**					**Deprivation gap (%) in 5-year survival^a^**				**5-year survival (%)**	
	**Patients diagnosed 1996–99**			**Average change every 5 years^b^**		**Patients diagnosed 1996–99**		**Average change every 5 years^c^**		**(period estimate, events in 2000–01)^d^**	
**Malignancy**	**No.**	**Survival (%)**	**(95% CI)**	**%**	**(95% CI)**	**Gap (%)**	**(95% CI)**	**%**	**(95% CI)**	**%**	**(95% CI)**
*Oesophagus*											
Men	12 814	7.6	(7.0, 8.2)	1.7**	(0.6, 2.8)	−1.9*	(−3.8, −0.1)	−1.4*	(−2.6, −0.1)	7.9	(6.9, 9.0)
Women	8219	7.2	(6.5, 8.0)	−0.3	(−1.7, 1.0)	−0.2	(−2.4, 2.0)	0.3	(−1.2, 1.8)	7.7	(6.5, 9.1)
											
*Stomach*											
Men	19 555	12.9	(12.3, 13.5)	2.0**	(0.9, 3.1)	−1.7	(−3.6, 0.1)	−0.8	(−1.9, 0.3)	14.7	(13.3, 16.1)
Women	10 618	14.0	(13.2, 14.9)	1.7*	(0.3, 3.2)	1.7	(−0.9, 4.3)	−0.5	(−2.0, 1.1)	18.0	(15.8, 20.2)
											
*Colon*											
Men	31 977	47.6	(46.8, 48.3)	5.6**	(4.2, 7.0)	−5.7**	(−8.0, −3.4)	−1.9*	(−3.4, −0.3)	51.7	(49.9, 53.5)
Women	32 243	47.4	(46.7, 48.1)	5.6**	(4.3, 6.8)	−7.3**	(−9.4, −5.1)	−2.2**	(−3.6, −0.8)	52.7	(50.7, 54.6)
											
*Rectum*											
Men	24 702	48.7	(47.8, 49.6)	7.4**	(5.8, 8.9)	−9.4**	(−12.0, −6.8)	−2.4**	(−4.1, −0.6)	50.6	(48.7, 52.5)
Women	17 264	51.3	(50.3, 52.4)	8.1**	(6.3, 10.0)	−8.3**	(−11.4, −5.2)	−2.5*	(−4.5, −0.5)	51.9	(49.4, 54.3)
											
*Pancreas*											
Men	8837	2.7	(2.3, 3.1)	0.1	(−0.6, 0.9)	−0.9	(−2.2, 0.4)	−0.6	(−1.5, 0.3)	2.4	(1.8, 3.1)
Women	9082	2.3	(1.9, 2.7)	−0.8*	(−1.4, −0.1)	1.1*	(0.1, 2.1)	0.7	(0.0, 1.4)	2.2	(1.7, 2.9)
											
*Larynx*											
Men	5666	64.3	(62.5, 66.1)	3.3*	(0.0, 6.7)	−17.2**	(−22.4, −11.9)	−3.7*	(−7.1, −0.2)	64.7	(60.6, 68.5)
											
*Lung*											
Men	67 862	6.0	(5.8, 6.3)	0.1	(−0.3, 0.5)	−1.4**	(−2.2, −0.7)	−0.3	(−0.7, 0.2)	6.5	(6.1, 7.0)
Women	39 455	6.5	(6.2, 6.8)	0.1	(−0.5, 0.7)	−0.6	(−1.6, 0.3)	0.0	(−0.6, 0.7)	7.3	(6.6, 7.9)
											
*Melanoma of skin*											
Men	7983	77.9	(76.5, 79.2)	4.0**	(1.6, 6.3)	−6.2**	(−10.2, −2.3)	−0.4	(−3.3, 2.6)	78.6	(76.0, 80.9)
Women	10 831	89.5	(88.6, 90.3)	0.3	(−1.2, 1.8)	−0.9	(−3.4, 1.6)	2.6**	(0.7, 4.4)	91.2	(89.3, 92.8)
											
*Breast*											
Women	125 093	79.8	(79.5, 80.1)	6.1**	(5.5, 6.7)	−5.8**	(−6.7, −4.8)	−0.1	(−0.8, 0.6)	81.4	(80.8, 82.0)
											
*Cervix*											
Women	10 344	65.5	(64.4, 66.6)	0.9	(−1.1, 2.8)	−5.1**	(−8.4, −1.7)	−0.4	(−2.4, 1.6)	65.7	(62.9, 68.4)
											
*Uterus*											
Women	16 549	75.8	(74.8, 76.7)	2.5**	(0.9, 4.1)	−4.2**	(−6.9, −1.6)	−0.8	(−2.7, 1.1)	77.1	(75.0, 79.1)
											
*Ovary*											
Women	20 177	38.1	(37.3, 38.9)	2.9**	(1.5, 4.3)	1.0	(−1.4, 3.5)	1.4	(−0.2, 3.0)	41.3	(39.3, 43.4)
											
*Prostate*											
Men	73 921	68.4	(67.7, 69.0)	15.9**	(14.8, 17.0)	−7.2**	(−9.0, −5.5)	−3.2**	(−4.5, −2.0)	71.1	(70.0, 72.2)
											
*Testis*											
Men	6153	96.5	(95.9, 97.0)	1.2	(−0.2, 2.5)	−1.3	(−3.3, 0.8)	1.8*	(0.2, 3.4)	97.9	(96.9, 98.5)
											
*Bladder*											
Men	29 252	66.5	(65.6, 67.4)	0.7	(−0.7, 2.2)	−5.7**	(−8.2, −3.1)	−0.9	(−2.5, 0.8)	66.0	(64.0, 68.0)
Women	11 345	56.3	(55.0, 57.6)	−0.8	(−3.0, 1.5)	−5.8**	(−9.5, −2.0)	−0.7	(−3.2, 1.8)	56.8	(53.4, 60.1)
											
*Kidney*											
Men	10 391	46.1	(44.8, 47.4)	4.5**	(2.1, 6.8)	−5.8**	(−9.5, −2.0)	−1.7	(−4.3, 0.9)	49.7	(46.4, 52.9)
Women	6191	45.7	(44.1, 47.3)	3.7*	(0.8, 6.7)	0.0	(−4.6, 4.6)	1.6	(−1.6, 4.8)	49.0	(44.4, 53.4)
											
*Brain*											
Men	6950	11.6	(10.7, 12.5)	−3.1**	(−4.7, −1.4)	2.6	(−0.1, 5.2)	2.5**	(0.6, 4.4)	11.3	(9.6, 13.2)
Women	5011	14.0	(12.9, 15.1)	−0.8	(−2.9, 1.2)	−1.4	(−4.6, 1.9)	−0.3	(−2.6, 2.0)	16.4	(13.6, 19.4)
											
*Non-Hodgkin Lymphoma*											
Men	13 952	51.3	(50.2, 52.4)	4.0**	(2.0, 5.9)	−7.3**	(−10.4, −4.1)	−1.5	(−3.8, 0.7)	54.5	(51.7, 57.3)
Women	12 281	52.7	(51.5, 53.9)	3.6**	(1.4, 5.8)	−5.4**	(−8.9, −1.9)	−0.4	(−2.8, 2.1)	55.9	(52.8, 58.8)
											
*Myeloma*											
Men	5300	25.6	(23.8, 27.4)	4.4**	(1.6, 7.3)	−4.8	(−10.0, 0.4)	−2.8	(−6.1, 0.4)	27.5	(24.1, 31.1)
Women	4754	23.8	(22.0, 25.6)	5.8**	(3.0, 8.7)	−7.7**	(−12.9, −2.6)	−5.6**	(−8.8, −2.4)	25.8	(22.4, 29.3)
											
*Leukaemia*											
Men	9966	39.7	(38.4, 41.0)	4.7**	(2.4, 7.0)	−4.4*	(−8.3, −0.5)	0.0	(−2.5, 2.6)	42.7	(39.3, 46.0)
Women	7715	35.9	(34.4, 37.3)	1.7	(−0.8, 4.2)	−2.5	(−6.7, 1.6)	1.8	(−0.9, 4.6)	39.4	(35.6, 43.1)

* Statistically significant at 5%;

** statistically significant at 1%.

a Average change in 5-year relative survival every 5 years over the period 1986–99, adjusted for deprivation (see text).

b Fitted difference in relative survival between most affluent and most deprived quintiles.

c Average change in deprivation gap every 5 years over the period 1986–99, adjusted for changes in survival over time (see text).

d Relative survival for patients diagnosed 1996–99 and alive during all or part of 2000–01 (see text).

). Brain tumour survival fell significantly. For women, survival increased for 13 of the 17 cancers examined, and the average increase every 5 years was significant for nine of these, but not for lung, melanoma, cervix or leukaemia. For cancers of the oesophagus, bladder or brain, survival was no better for women diagnosed in 1996–99 than for those diagnosed in 1986–90, and survival was significantly lower for pancreatic cancer.

Survival for patients living in the poorest one-fifth of electoral wards of England and Wales when diagnosed during 1996–99 was lower than for those in the richest fifth of wards for 28 of the 33 cancer–sex combinations (Table 2). For 11 cancers in men and nine in women, this deprivation gap in survival was statistically significant.

For 12 of the 16 cancers examined in men, the deprivation gap in survival between rich and poor was wider for those diagnosed during 1996–99 than for those diagnosed during 1986–90. The average increase every 5 years in the deprivation gap over that period was itself statistically significant for cancers of the oesophagus, colon, rectum, larynx and prostate, and for laryngeal cancer the deprivation gap in survival reached 17% for men diagnosed during 1996–99. By contrast, there was a significant reduction in the deprivation gap for testicular and brain tumours.

For women, the deprivation gap in survival also increased during the 1990s, but less markedly than for men. For nine of the 17 cancers examined, the deprivation gap was wider for women diagnosed during 1996–99 than 1986–90. The average increase every 5 years in the deprivation gap was significant for cancers of the colon and rectum, and for myeloma. By contrast, there was a significant reduction in the deprivation gap for melanoma of the skin, and it also became slightly less marked for cancers of the oesophagus, pancreas, ovary and kidney, and the leukaemias (Table 2).

Five-year survival for lung cancer patients diagnosed during 1996–99 was 6% in men and women, not significantly better than for patients diagnosed a decade or so earlier. Survival among men was significantly lower for the poor than the rich (deprivation gap −1.4%), a wider gap than for men diagnosed 1986–90, although the 5-yearly increase in the gap was not itself significant. The deprivation gap in survival for women diagnosed during 1996–99 was small, and unchanged from a decade earlier.

Breast cancer survival at 5 years in women diagnosed during 1996–99 was 80%, a rapid and significant average increase of 6.1% every 5 years since 1986–90. The deprivation gap in survival was −5.8% (95% CI −6.7 to −4.8%), no different to that for women diagnosed during 1986–1990.

Prostate cancer survival at 5 years among men diagnosed during 1996–99 was 68%, a remarkable average increase, after adjustment for deprivation, of 15.9% every 5 years (95% CI 14.8–17.0%) from 43% for men diagnosed during 1986–90. The deprivation gap in survival steepened significantly by about −3% every 5 years during the 1990s, reaching −7.2% for men diagnosed during 1996–99.

**Figure 2:**
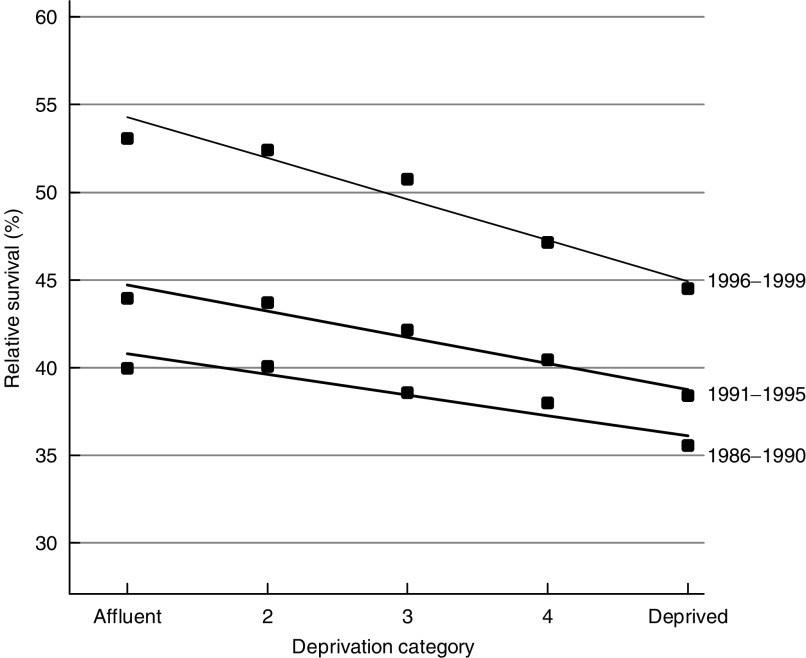
Five year relative survival (%) by deprivation category and calendar period of diagnosis, England and Wales, men with cancer of the rectum diagnosed in successive calendar periods 1986–99 and followed up to 2001.

Five-year survival from colon cancer increased by about 6% every 5 years in both sexes, to about 47–48%, and rectal cancer survival rose by about 8% every 5 years, to 49% for men diagnosed during 1996–99 (Figure 1) and 51% in women. The deprivation gap in 5-year survival became significantly steeper during the 1990s for both cancers and in both sexes, reaching −6% to −7% for colon cancer and −8% to −9% for rectal cancer (Figure 2

).

For brain tumours, 5-year survival fell in both sexes to 12–14% for those diagnosed 1996–99. For men, the average decline of 3.1% every 5 years was statistically significant at the 1% level. This anomalous trend was accompanied by a positive deprivation gradient of borderline significance (2.6%; 95% CI −0.1% to +5.2%).

**Figure 3:**
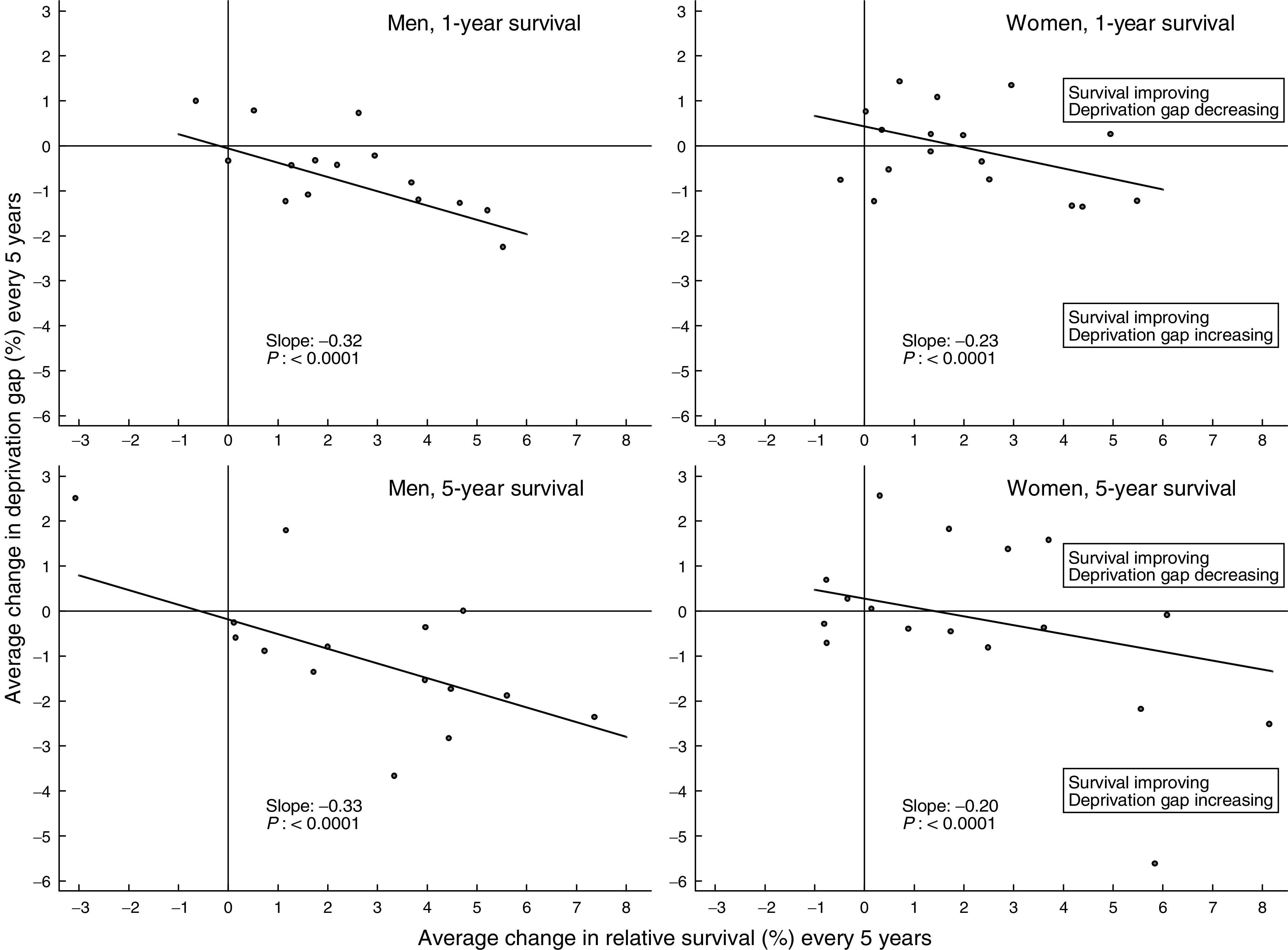
Average change every 5 years in relative survival (%) and in the deprivation gap in survival (%): England and Wales, 1- and 5-year survival, selected cancers, men and women diagnosed during the period 1986–99 and followed up to 2001. Prostate cancer was excluded from the regression and the scatter-plot (see text).

Improvements in 1- and 5-year survival for the 20 most common cancers were significantly associated with an increase in the deprivation gap in both sexes (Figure 3

: each data point represents a different cancer). The association is more marked for men, but all four associations are statistically significant. Prostate cancer was excluded from this analysis because of the outlying rate of increase in 5-year survival (15.9% every 5 years), but the slope of the regression was only marginally steeper as a result.

The results suggest that for the 16 cancers examined in men, an increase of 5% in the national average 5-year survival rate during the 1990s was typically associated with an increase of about 1.5% in the deprivation gap in survival between rich and poor.

### Period analysis

Survival estimates based on period analysis for 2000–01 are slightly higher for most cancers than conventional survival estimates for patients diagnosed during 1996–99 (Table 2). These predictions generally confirm the improvements observed over the period 1986–99.

## DISCUSSION

Cancer survival improved during the 1990s for almost all the 20 common cancers examined here. Period survival estimates based on events during 2000–01 were higher for most cancers than conventional estimates for patients diagnosed during 1996–99, suggesting that survival rates are likely to continue rising for the next few years. For many cancers, however, survival improved more for patients living in affluent wards than for those living in deprived wards. As a result, the existing deprivation gap in survival between rich and poor widened during the 1990s.

There were some notable exceptions to these patterns. Average survival from brain tumours for both men and women fell slightly but steadily during the 1990s. One possible explanation would be greater use of cerebral CT or MRI imaging for affluent patients. Cerebral tumours might then have been diagnosed more often in affluent patients presenting with rapidly fatal intracranial events that might previously have been diagnosed (and certified without post-mortem) as a cerebrovascular accident. This explanation is supported by the fact that incidence in the most affluent group rose more than in deprived groups between 1986 and 1999, and survival in the first 6 months after diagnosis fell more (data not shown).

The reduction in the deprivation gradient for testicular cancer survival suggests a ceiling effect, since the national average 5-year survival estimate for men diagnosed during 1996–99 was 96%, and any further increase in survival would be expected to affect men in the less affluent groups, whose survival had been lower. The reduction in the deprivation gap for women with melanoma (national average 5-year survival 89%) may have a similar explanation.

The extraordinary deprivation gap in survival for men diagnosed with laryngeal cancer during 1996–99 was significantly wider than for those diagnosed in the early 1990s or in previous decades (Coleman *et al*, 1999), yet there is no evidence of differential trends in incidence between socioeconomic groups that might have suggested drift in the type of malignancy being recorded (data not shown).

Survival from cancers of the rectum and colon increased more rapidly during the 1990s in both sexes than during the 1970s or the 1980s (Coleman *et al*, 1999). These trends are consistent with the continuing decline in mortality and the steady rise in incidence (Quinn *et al*, 2001). The increase in survival may be attributable to improvements in surgery (McArdle and Hole, 2002) and reduction in operative mortality (Mitry *et al*, 2002). Taken together, these trends suggest that improvements in survival may be attributable both to earlier diagnosis and to improved treatment. Improvements in survival were notably faster for the more affluent groups in both sexes, however: the deprivation gap in 5-year survival for rectal cancer increased significantly to 8% for patients diagnosed 1996–99 and 6% for colon cancer. Adverse prognostic factors such as proximal subsite and mucin-producing adenocarcinoma are not more common in lower socioeconomic groups (Lyratzopoulos *et al*, 2003), and differences in stage at diagnosis do not appear to explain the survival gradient (Schrijvers *et al*, 1995). This suggests that the most deprived patients may not have benefited equally from advances in early diagnosis and treatment. Incidence trends for rectal cancer during the 1990s were similar for all deprivation groups, but the overall increase in colon cancer was smaller in the least affluent group (data not shown): colonoscopic polypectomy may be less accessible to the least affluent patients, but it is unclear if that alone could account for the differences in incidence, and only invasive malignant tumours were included in this study. A large study of colorectal cancer patients hospitalised in the US Veteran Administration health care system during the period 1987–98 did not show the survival differences between black and white patients consistently reported from the SEER program of population-based registries. The authors suggest that this may be attributable to the VA being an equal access system, without financial and health insurance barriers to care (Rabeneck *et al*, 2003).

The extremely rapid increase in prostate cancer survival reflects the increase in diagnosis and treatment of asymptomatic prostatic cancers as a result of increasingly widespread use of prostate-specific antigen (PSA) tests during the 1990s (Evans and Møller, 2003). Many of these tumours would never have been diagnosed in life, and PSA testing has led to a rapid increase in recorded incidence and survival rates in many countries (Coleman *et al*, 2003). The trend in prostate cancer survival in the population is not an artefact, however: it simply reflects the rapid shift in the biological and clinical spectrum of prostate tumours that are now being diagnosed as a result of new diagnostic techniques such as PSA. The concurrent widening of the deprivation gap in survival suggests that access to PSA tests has been more widespread among men in affluent groups than in deprived groups. It is open to question whether the trend in survival during the 1990s represents an improvement for individual men with very early prostate cancer.

### Bias and artefact

It is unlikely that the observed patterns or trends in the deprivation gap in survival between rich and poor can be explained by bias or methodological factors. The gradients for patients diagnosed during 1996–99 are flatter than if we had not taken account of the widening socioeconomic gradient in background mortality (Department of Health, 1999) by constructing separate deprivation-specific life tables for both the early and the late 1990s (Coleman *et al*, 1999). Increases in deprivation gradient during the 1990s are therefore smaller than they would have been without this adjustment.

Exclusion from the analyses of patients whose duration of survival was unknown (DCO cases) cannot explain the deprivation gradients, because for each cancer the proportion of DCOs was very similar in all deprivation groups (data not shown).

Selective failure to register cancer patients who survive longer than average would produce lower survival (and incidence) rates, but for such a bias to give rise to artefactual deprivation gradients in survival, it would have to affect the poor more than the rich. We simulated the impact on national survival estimates of all the cancer registries in England and Wales having selectively failed to register up to 30% of longer-term survivors in the most deprived group, with lower proportions in intermediate groups and assuming complete registration in the most affluent group. For most cancers, such selective under-registration would have to exceed 30% in order to account for the observed survival gradients (data not shown). This is not likely. The absence of any deprivation gradient in DCOs makes it an even less plausible explanation, since DCOs also reflect inefficiency in the registration of cancer patients during life (Jensen *et al*, 1991).

Full cohort estimates of survival for patients diagnosed in 1996, for whom at least 5 years' follow-up were available, were very similar to estimates using all the available data for patients diagnosed 1996–99 (complete analysis).

### Public health implications

In 1995, an Expert Advisory Group on Cancer to the Chief Medical Officers in England and Wales noted regional disparities in cancer survival, and recommended the reorganisation of cancer treatment services to ensure that all patients had access to ‘*a uniformly high quality of care .. wherever they may live, to ensure the maximum possible cure rates and best quality of life*’ (Calman-Hine report) (Expert Advisory Group on Cancer, 1995). In December 2001, however, the National Confidential Enquiry on Perioperative Deaths (NCEPOD) reported wide variation in hospital caseloads and experience, inadequate staging by clinicians, insufficient reporting of stage by pathologists and poor compliance with treatment guidelines for cancer patients operated as recently as 1999–2000 (NCEPOD, 2001).

Our analyses confirm that cancer survival in England and Wales improved significantly during the 1990s. They also show that improvements in survival were usually greater for those living in affluent areas than those in deprived areas, even after correction for the widening differences in overall mortality between rich and poor. The results show a link between these trends, for the first time to our knowledge. For the 20 most common cancers in England and Wales, overall increases in survival during the 1990s were significantly associated with a widening deprivation gap in survival. Breast cancer and melanoma in women, and testicular cancer in men were important exceptions to this pattern.

Such observations suggest the influence of delay or less effective access to diagnosis and treatment for patients living in more deprived areas. Lower socioeconomic groups tend to use NHS services less in relation to need: this may reflect barriers to access such as transport or time constraints, and differences in knowledge or beliefs about the need for medical attention, but patients from higher socioeconomic groups may also communicate more effectively with the medical profession to obtain health care (Dixon *et al*, 2003).

The NHS Cancer Plan, published in September 2000, aims to reduce inequalities in cancer survival between rich and poor (Department of Health, 2000), and the government has set challenging targets for the NHS to reduce health inequalities (Department of Health, 2003a).

Cancer patients diagnosed in 2000 or later could not be included in these analyses, although period estimates suggest some continuing improvement in survival. Up-to-date evidence on survival by socioeconomic group will soon be needed to evaluate whether the NHS Cancer Plan has helped to improve cancer survival and to reduce inequalities in survival (Department of Health, 2003b). The results published here underline the importance of the NHS Cancer Plan being made to work.
